# The Wound Healing Effects of Herbal Cream Containing *Oliveria Decumbens* and *Pelargonium Graveolens* Essential Oils in Diabetic Foot Ulcer Model

**Published:** 2018-01

**Authors:** Mohaddese Mahboubi, Mohsen Taghizadeh, Tahereh Khamechian, Omid Reza Tamtaji, Rasoul Mokhtari, Sayyed Alireza Talaei

**Affiliations:** 1 Microbiology Department, Medicinal Plant Research Center of Barij, Kashan, Iran;; 2 Research Center for Biochemistry and Nutrition in Metabolic diseases, Kashan University of Medical Sciences, Kashan, Iran;; 3 Department of Pathology, Faculty of Medicine, Kashan University of Medical Sciences, Kashan, Iran;; 4 Physiology Research Center, Kashan University of Medical Sciences, Kashan, Iran

**Keywords:** Herbal, Oliveria decumbens, Pelargonium graveolens, Diabetic foot ulcer

## Abstract

**BACKGROUND:**

The number of diabetic patients in adult population is increasing. All this population are at risk of developing diabetic foot ulcers (DFUs) that are associated with unwanted ailments and high mortality. In spite of current therapies for DFUs, further therapies are needed to help the patients.

**METHODS:**

The efficacy of herbal cream containing *Pelargonium graveolens* and *Oliveria decombens* essential oils was evaluated topically for treatment of DFUs in rat animal model in comparison with two other herbal formulas containing each essential oil alone, placebo (the basic formula without active ingredients) and normal saline as control groups. After anesthesia of diabetic rats (n=75) induced by streptozotocin (STZ), diabetic wounds were visible on the hind dorsal surface **of the foot**. The treatments were initiated on Day 1 and repeated 3 times a day for thirteen consecutive days. On day 1, 3, 5, 8 and 13, the wound sizes were determined and assessed histologically.

**RESULTS:**

Three herbal formulations reduced the size of wounds in rats with DFUs, while the cream containing combined herbals of *O. decumbens* and *P. graveolens* essential oils had the highest tissue repair in DFU rat models.

**CONCLUSION:**

Due to better wound healing effects of combined herbal cream containing *O. decumbens* and *P. graveolens* essential oils, it can be recommended in treatment of DFUs.

## INTRODUCTION

The number of diabetic patients in adult population was estimated to be 382 million in 2013.^1^ Twenty-five percent of diabetic populations are at risk of developing a diabetic foot ulcer (DFU).^2^ Eighty-five percent of foot diabetic ulcers may cause amputation. DFUs occurs as a result of peripheral nephropathy ^3^ and ischemia from peripheral vascular diseases.^4^ The management of DFUs includes debridement and several healing process.^5^ If these wounds are infected, the appropriate antibiotic therapies are administered by clinicians.^6^ Further preventive treatments and drugs are needed even though there are several therapies can assist in the overall healing process of wounds in DFUs.^7^

Medicinal plants are old promising resources for exploring the new agents for treatments of different ailments, such as diabetic related ailments.^8^^-^^10^ Two important plants among the medicinal plants that are used for the treatment of skin diseases are *Pelargonium graveolens* and *Oliveria decumbens*. *P. graveolens* essential oil is utilized in many traditional systems as anti-allergic, diuretic, tonic, anti-diabetic.^11^^-^^13^ The prominent property of the aerial parts of Pelargonium genus is its curative and palliative effects in wound healing.^12^^,^^14^^,^^15^ In Iranian Traditional Medicine, *O. decumbens* essential oil is used for the treatment of indigestion, diarrhea, abdominal pains, fever and infectious diseases.^16^

The typical microorganism that is usually isolated from the wounds of DFUs is Gram positive cocci especially *Staphylococcus aureus*.^17^ The antibacterial activity of *O. decumbens*^16^^,^^18^ and *P. graveolens* ^19^^,^^20^ essential oils were confirmed against clinical isolates of *S. aureus* by others.^12^^,^^14^^,^^15^ So due to the wound healing,^12,14,15^ hypoglycemic and antioxidant effects^11^ of *P. graveolens* essential oil,^16^^,^^18^ the antimicrobial property of *O. decumbens*,^16^^,^^18^^-^^20^ and the utilization of two essential oils together may have some beneficial effect in the management of DFUs, this research evaluated the therapeutic efficacy of cream containing *O. decumbens* and *P. graveolens* essential oils on wound healing in rat animal model with DFUs.

## MATERIALS AND METHODS

Flowering aerial parts of *O. decumbens* and *P. graveolens* were collected from Research farm of the Medicinal Plant Research Center, Barij (Kashan, Iran) in June 2014. The voucher specimens were identified and deposited in the Herbarium of the Department of Agriculture, Medicinal Plants Research Center, Barij, Kashan Iran. For extraction of essential oils, 100 g of each plant was milled and mixed with 1200 ml water and was boiled in a Clevenger type apparatus for 3 h. The oils were separated and kept in cooled place until analysis.^21^

The chemical compositions of essential oils were analyzed by GC and GC-MS. The GC and GC-MS assays were carried on Agilent technology model (6890) equipped with capillary column of HP-1MS (30 mm×0.25 mm, film thickness 0.25 μm). The oven temperature program was initiated at 40°C and was held for 1 min, then it was raised to 230°C at a rate of 3°C/min held for 10 min. Helium was utilized as the carrier gas at a flow rate of 1.0 ml/min. The detector and injector temperatures were 250 and 230°C, respectively. Retention indices (RI) were calculated for all components using a homologous series of alkanes injected in conditions equal to the conditions of the sample. The results were interpreted by computer search using libraries of Wiley275.L and Wiley7n.1, as well as comparisons of the fragmentation pattern of the mass spectra with data published in the literature.^22^ The essential oils were formulated as topical cream in the Research Center of Barij Essence Pharmaceutical Company, Kashan, Iran.^23^

Adult male Wistar rats weighing 200-220 g were bought from Kashan University of Medical Sciences (Kashan, Iran). They have free access to food and water ad libitum. The experimental subjects were kept in a single holding room and housed in a constant temperature of 21±2^o^C, humidity of 55±5% and under 12-h light/dark cycles. All experiments were carried out in accordance with the UK Animals Scientific Procedures Act 1986 (86/609/EEC).

Diabetes was induced in Wistar rats by intra-peritoneal injection of 65 mg/kg of Streptozotocin (Sigma, USA). After four weeks, the diabetic rats with glucose level higher than 300 mg/dL were utilized for wound induction. On day 0 (the day of wound induction), each rat was anesthetized with intra-peritoneal injection of 1.43 mg/kg diazepam (Khemidaru, Iran) and 13 mg/kg ketamine (10%) (Alfasan, Woerden-Holland). A rectangular layer (2×5 mm) of skin on the hind dorsal surface of foot was removed. A total of 75 rats were divided into five groups (n=15 in each group) including (i) *O. decumbens* oil 1% cream (O), (ii) *P. graveolens* oil 1% cream (P), (iii) *O. decumbens* 1% and *P. graveolens* 1% oils cream (OP), (iv) Placebo (the basic formula without active ingredients) (PL), (v) Normal saline (S). 

The treatments were initiated on day 1 and repeated three times in a day for thirteen consecutive days. The wound sizes were measured on day 1, 3, 5, 8 and 13 by digital camera by the same investigator and the results of pictures were analyzed using Image Analysis Computer software (Scion Image Software, USA). Multilevel statistical model (SPSS version 11.0, Chicago, IL, USA) was used to compare the results on days 3, 5, 8 and 13 regarding the baseline (day 1). All statistical tests were two sided, and statistical significant was set at *p*<0.05.^24^

In each group, rats were randomly selected and after sacrificing the rats, their tissues were separated. Then, the tissues were preserved and fixed in 10% formalin. The fixed sections were sliced and stained with hematoxylin and eosin (H&E). The vascularization, epidermis repair and aggregation of inflammatory cells were scored as 0=not good, 1=good, and 2=very good. The sum of scores for vascularization, epidermis repair and aggregation of inflammatory cells were estimated for each rat^25^ and were analyzed by Graph Pad prism 6. The higher score exhibited the better tissue regeneration.^26^

## RESULTS

The chemical attributes of essential oils used in our formulations were investigated. GC and GC-MS analysis of *P. graveolens* essential oil demonstrated the presence of β-citronellol (34.4%), geraniol (10.1%) and phenyl ethyl alcohol (9.4%) as the main components of essential oil. Thymol (50.1%), γ-terpinene (20.7%), croweacin (5.3%) and sabinene (1.5%) were the main components of *O. decumbens* essential oil. The wound healing effects of different essential oils formulation were evaluated against the DFUs and the ulcer areas were measured in different groups (Table 1). As illustrated in Table 1, there was no significant difference between Group I, II, III (*p*>0.05) on the base of ulcer area at time of zero (day 1). The diameter of ulcer areas was reduced time dependently in all groups (Table 1). 

**Table 1:** Ulcer area measurement (cm) in rat diabetic model.

**Day**	**1st**	**3rd**	**5th**	**8th**	**13th**
**Groups**					
I	0.72	0.66	0.54	0.17	0.04
II	0.68	0.67	0.59	0.27	0.13
III	0.63	0.57	0.55	0.15	0
IV	0.64	0.6	0.55	0.38	0.24
V	0.64	0.64	0.53	0.39	0.27

I: *O. decumbens* essential oil; II: *P. graveolens* essential oil; III: *O. decumbens* plus *P. graveolens *essential oils; IV: Placebo; V: Normal Saline

After 13 days, the means of ulcer area (mm) reduced from 0.72 to zero in Group III, while these reductions were 0.72 to 0.04 for Group I and 0.68 to 0.13 for Group II. Among the three groups above, the wound healing effects of Group III was significantly better than the other two groups (*p*<0.05). At day 13, there was no significant difference between groups V and IV on the base of ulcer area but there was a significant difference between the three other groups especially Group II.

**Fig. 1:**
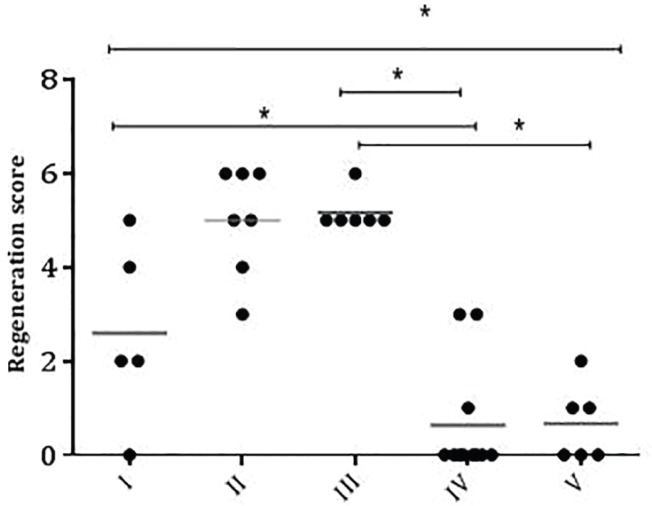
The regeneration scores for different groups of diabetic foot ulcers. I: *O. decumbens* essential oil; II: *P. graveolens* essential oil; III: *O. decumbens* plus *P. graveolens *essential oils; IV: Placebo; V: Normal Saline.

The histopathological analysis of tissues in various groups showed the higher regeneration score for Group III, followed by Group II. The worst score was for Group IV, followed by Groups V and I. There was no significant difference between Groups II and III as well as between Groups I and V (Figure 1). There was a significant difference between Groups I and IV. Indeed, Group I was a little better than placebo (IV) and control (V) groups in regenerating tissues with DFUs. Groups II and III had the highest scores in regeneration of tissues from diabetic foot ulcers. 

## DISCUSSION

In this study, we evaluated the efficacy of new topical herbal cream containing *P. graveolens* and *O. decumbens* essential oils in improvement of DFUs in animal rat models. As demonstrated in the results of our study, this cream has a valuable efficacy in reducing the ulcer area and regeneration of tissues in diabetic foot ulcers. Indeed, the potency of this topical cream in reducing of ulcer area or wound size is related to the main active components of formulation such as *P. graveolens* and *O. decumbens* essential oils. 

Although, many pharmacological activities of *O. decumbens* such as antibacterial, antifungal activities^16^^,^^18^ were confirmed by others, our results confirmed the anti-ulcer effect of *O. decumbens *essential oil for the first time. Thymol has been confirmed as the main component of *O. decumbens* essential oil which protects the stomach from ulcer by regulation of matrix metalloprotein 9 activity.^21^ The anti-inflammatory and wound healing effects of thymol has also been confirmed.^27^ So, the cream containing *O. decumbens* essential oil may have other pharmacological effects other than anti-ulcerogenic and regenerative effects such as antimicrobial and anti-inflammatory effects in animal models. More pharmacological studies are needed to confirming these effects.

The combination of *P. graveolens* and *O. decumbens* essential oils had anti-ulcerogenic effects, a little higher than that of each cream containing each single essential oil. So, the combination of two essential oils can increase its anti-ulcerogenic effect after a while. The anti-inflammatory effects of *P. graveolens* essential oil have been confirmed in many studies.^23^^,^^28^^,^^29^ Other researchers showed that *P. graveolens* essential oil suppressed nitric oxide and prostaglandin E2 in a dose dependent manner, demonstrating the efficacy of *P. graveolens* essential oil in inflammation associated disorders.^29^

The anti-inflammatory effects and tissue regeneration of our herbal cream may help in reducing inflammation; the second stage wound healing and increasing the proliferation and remodeling of wounds. Therefore, the various pharmacological effects of two essential oils in the designed herbal cream can help for proper regeneration and wound healing when the combined cream is applied than when *P. graveolens* essential oil cream is applied alone in ulcer treatment and tissue regeneration. However, the tissue regeneration ability of *O. decumbens* cream was lower than that of creams containing *P. graveolens* essential oil. 

Diabetes treatments and its complications impose a major economic burden to the patients and health facilities due to the severity of the disease.^29^ Therefore, choosing the cheap effective treatment may reduce the costs of diabetes treatments. The designing of this herbal cream containing only two percent essential oils in basic formula involves lower cost for diabetic patients with DFUs. Because the combination of *P. graveolens* and *O. decumbens* essential oils had the best anti-ulcerogenic effect and tissue regeneration, it is recommended for treatment of DFUs. Further studies in order to evaluate the precise mechanism of action of tissue regeneration and anti-ulcer effects of these new formulation cream seem necessary.
